# Complex relationship between amino acids, fitness and food intake in *Bombus terrestris*

**DOI:** 10.1007/s00726-021-03075-8

**Published:** 2021-09-29

**Authors:** C. Ruth Archer, Johannes Fähnle, Maximilian Pretzner, Cansu Üstüner, Nina Weber, Andreas Sutter, Vincent Doublet, Lena Wilfert

**Affiliations:** 1 Institute of Evolutionary Ecology and Conservation Genomics, University of Ulm, Albert-Einstein-Allee 11, 89081 Ulm, Germany; 2 School of Biological Sciences, University of East Anglia, Norwich, UK; 3 College of Life and Environment Sciences, University of Exeter, Tremough Campus, Penryn, TR10 8FL UK

**Keywords:** *Bombus*, Geometric framework of nutrition, Intake array, Trade-off

## Abstract

The ratio of amino acids to carbohydrates (AA:C) that bumble bees consume has been reported to affect their survival. However, it is unknown how dietary AA:C ratio affects other bumble bee fitness traits (e.g., fecundity, condition) and possible trade-offs between them. Moreover, while individual AAs affect phenotype in many species, the effects of AA blend on bumble bee fitness and food intake are unclear. We test how the AA:C ratio that bumble bees (*Bombus terrestris*) consume affects their condition (abdomen lipid and dry mass), survival following food removal, and ovarian activation. We then compare ovarian activation and food intake in bees fed identical AA:C ratios, but where the blend of AAs in diets differ, i.e., diets contained the same 10 AAs in an equimolar ratio or in the same ratio as in bee collected pollen. We found that AA:C ratio did not significantly affect survival following food removal or ovarian activation; however, high AA intake increased body mass, which is positively correlated with multiple fitness traits in bumble bees. AA blend (i.e., equimolar versus pollen) did not significantly affect overall ovarian activation or consumption of each experimental diet. However, there was an interaction between AA mix and dietary AA:C ratio affecting survival during the feeding experiment, and signs that there may have been weak, interactive effects of AA mix and AA:C ratio on food consumption. These results suggest that the effect of total AA intake on bumble bee phenotype may depend on the blend of individual AAs in experimental diets. We suggest that research exploring how AA blend affects bumble bee performance and dietary intake is warranted, and highlight that comparing research on bee nutrition is complicated by even subtle variation in experimental diet composition.

**Supplementary Information:**

The online version contains supplementary material available at 10.1007/s00726-021-03075-8.

## Introduction

How much energy an individual consumes, as well as the blend of nutrients that provides this energy, affects the expression of key fitness traits (Simpson and Raubenheimer 2012). For example, in many species low protein, high carbohydrate diets improve lifespan (Nakagawa et al. 2012). Moreover, different fitness traits may be optimised on different nutrient blends. For example, while low protein consumption typically extends lifespan in omnivorous insects, female fecundity is often maximised on higher protein intake (e.g. Jensen et al. 2015; Lee et al. 2008; Maklakov et al. 2008; Rapkin et al. 2017). This drives a dietary mediated trade-off between these traits, because females cannot invest maximally in both traits at the same time (Lee et al. 2008; Maklakov et al. 2008). Understanding how diet affects fitness, and trade-offs between fitness traits  is a key aim in evolutionary biology (Moatt et al. 2020). Moreover, characterising a species’ dietary demands sheds light on its wider ecology (Simpson and Raubenheimer 2012) and nutritional physiology is a key tool in conservation (Madliger et al. 2018). For this latter reason in particular, a great deal of research has characterised how nutrients affect bumble bee foraging behaviour, and fitness (Grund-Mueller et al. 2020; Leonhardt and Blüthgen 2012; Stabler et al. 2015; Vaudo et al. 2016a,b, 2018,2020).

Many bee species are experiencing severe declines with for example, almost one in four bumble bee species on the IUCN Red List in decline (Cameron and Sadd 2020). Agricultural intensification is a key driver of these losses, in part because associated land-use changes may reduce forage diversity, quality and abundance (Cameron and Sadd 2020; Wilfert et al. 2020). Poor forage may lead to malnutrition and increase bee vulnerability to other environmental drivers of pollinator loss, e.g., pathogens or toxins (Goulson et al. 2015). Understanding how nutrition affects bumble bee phenotype is a key step in determining how well bees meet their dietary demands in nature and rectifying any deficiencies (Vaudo et al. 2020, 2015). Building this understanding seems relatively simple, because foraging bumble bees only retrieve two types of food—nectar and pollen (Nicolson 2011; Vaudo et al. 2015). Nectar is primarily water and carbohydrate, but also contains amino acids (AAs), inorganic ions and secondary metabolites, while pollen principally contains proteins, lipids and carbohydrate as well as free AAs (Nicolson 2011; Roulston and Cane 2000). Nutrient composition of pollen and nectar varies between plant species and this variation affects bee behaviour—nectar sugars (Abrol and Kapil 1991), pollen AAs (Leonhardt and Blüthgen 2012), protein and lipids (Vaudo et al. 2020) all affect bee foraging decisions. Bee diets are evidently not quite as simple as they appear on first  sight.

Research testing how nutrients jointly affect bumble bees has focussed on the primary components of bee diets: AAs or protein, lipids and carbohydrate. This work has shown consistent effects of these nutrients on bumble bee fitness. Colony intake rate of protein, lipid, and carbohydrate correlates with *Bombus impatiens* colony growth and reproductive output (Vaudo et al. 2018)*. B. terrestris* fed a range of diets varying in their protein:lipid ratios (P:L) survived best when they consumed a 10:1 ratio (Vaudo et al. 2016b). In general, high AA or protein intake is associated with poor survival in honey and bumble bees (Archer et al. 2014a; Paoli et al. 2014; Stabler et al. 2015). However, the costs of high AA or protein intake seem to depend on the source of dietary nitrogen: studies report poor survival in bees fed diets rich in an equimolar mixture of AAs (Stabler et al. 2015), while a study supplementing pollen with AAs in the ratios that they appear in pollen did not see such negative effects (Ruedenauer et al. 2020).

The balance of nutrients in experimental diets also affects bumble bee food intake. *B. impatiens* able to self-select their food consumption from pairs of nutritionally modified pollens preferentially consumed pollens with a P:L ratio between 5:1 and 10:1 (Vaudo et al. 2016a), while in the field *B. impatiens* collected pollen with an average P:L ratio of 4:1 (Vaudo et al. 2018). However, once again, the source of dietary nitrogen appears to affect nutrient regulation strategies: *B. terrestris* able to self-regulate their nutrient intake chose a 1:149 (w/w) protein to carbohydrate (P:C) ratio when fed diets containing casein as a protein source, but chose a 1:560 (w/w) essential amino acid to carbohydrate (EAA:C) ratio when fed an equimolar AA mix (Stabler et al. 2015).

These results suggest that the effect of dietary nitrogen on bees depend on the protein source. This is perhaps not surprising given that even individual AAs can have pronounced effects on fitness. For example, altering just dietary methionine affects *Drosophila melanogaster* lifespan significantly (Troen et al. 2007). AAs are clearly important in bees. The AA profile of pollens determines how much pollen bees need (Nicolson 2011). At least 10 AAs are vital for growth and survival in honey bees (*Apis mellifera*) (de Groot 1952), but—as highlighted above—excessive AA intake can reduce survival in *B. terrestris* (Stabler et al. 2015) and honey bees (Archer et al. 2014a; Paoli et al. 2014). AAs also influence bee foraging decisions—bumble bees may prefer pollens rich in EAAs (Leonhardt and Blüthgen 2012; but see Ruedenauer et al. 2020) and AA receptors at the tip of bumble bee antennae may help them assess the AA content of foods prior to consuming them (Ruedenauer et al. 2019). Understanding how AAs affect phenotype could arguably be considered the foundation of bee nutritional ecology but building this understanding is challenging—testing how 20 AAs jointly affect bee fitness is a 20 dimensional problem (discussed in Piper et al. 2017). To date, research testing how AAs affect bees has either tested the effects of one AA at a time (de Groot 1952) or grouped AAs together (Archer et al. 2014a; Grund-Mueller et al. 2020; Paoli et al. 2014; Stabler et al. 2015) and so it is unclear how AAs interact with one another to influence fitness. Given accumulating data suggesting that the blend of AAs in experimental diets might affect outcomes, work explicitly testing how AA blend affects bee phenotype and nutrient regulation is needed.

The aims of this manuscript are twofold. First, we test how the ratio of AA:C that bumble bees consume affect multiple traits that indicate individual bee condition—body composition (abdomen lipid and dry mass), survival during the feeding experiment and following food removal, and ovarian activation. Research on how nutrition affects bees typically tests how diet affects survival (Archer et al. 2014a,b; Paoli et al. 2014; Stabler et al. 2015; Vaudo et al. 2018) and sometimes reproduction (e.g. Altaye et al. 2010; Grund-Mueller et al. 2020). By assaying dietary effects on multiple traits here, we aim to capture a more complete picture of how AAs and carbohydrate jointly affect *B. terrestris* fitness and identify any dietary mediated trade-offs between the traits assayed. Second, we test if the AA blend in experimental diets influences the relationship between AA:C ratio and ovarian activation (the trait we predicted a priori most likely to be affected by AA mix), as well as survival during the feeding experiment and how bees regulate their nutrition when constrained to a single nutritionally imbalanced diet. To achieve this, we fed bees diets that contained the same 10 AAs but in different ratios—first, an equimolar ratio and second, an AA blend comparable to that in bee collected pollens (hereafter, pollen mix). We find that as the AA portion of experimental diets increased abdomen mass (excluding lipids) increased too, but ovarian activation was unaffected. Effects of dietary AA:C ratio on survival following food removal were non-significant, but there was a significant interaction between dietary AA:C ratio and AA mix (i.e., equimolar / pollen) affecting survival during the experiment. AA mix did not significantly affect ovarian activation or total food intake—however, trends in the data suggest that AA mix interacted with AA:C ratio to affect dietary intake. Accordingly, we suggest that future bee research should standardise experimental AA mixes to generate comparable outputs.

## Materials and methods

### General methods

#### Animals and husbandry

*Bombus terrestris* is an economically important bumble bee that survives well in cages and so is amenable to dietary manipulation work. Seven *B. terrestris* colonies were purchased from Biobest Belgium N.V and maintained at 26 °C with *ad libitum* irradiated pollen (Biobest, gamma radiation) and sugar water—Invertbee feed sugar (*BelgoSuc*) diluted 1:1 with water (Manley et al. 2017) under red light. To test if colonies were healthy, four bees were collected at random from each of the experimental colonies and their faeces were microscopically examined for *Nosema bombi, Crithidia spp and Apicystis bombi.* After confirming that the sampled bees were free from these pathogens, the five largest colonies were used for experimental work. Experimental bees were housed in micro-colonies of five individuals in a plastic box (12 × 9 × 6 cm) with holes in the lid for ventilation, lined with *circa* 1 cm of cat litter (Cat’s Best by J. Rettenmair & Söhne GmbH & CO KG) to regulate humidity, and containing a petri-dish lid that held experimental diets in place. One bee from each of the five main experimental colonies was allocated to each experimental micro-colony. If a bee died on the first day of the experiment, it was replaced with a randomly selected replacement individual and that death was not included in survival analyses—we attributed this mortality to a handling death rather than an effect of experimental diet.

#### Diet making and feeding

Diet making procedures follow those used by Stabler et al. (2015) but here, two AA mixes rather than one were used to create experimental diets. The first was an equimolar AA mix, where each of ten EAAs used in previous experiments on bumble bees was added to a 0.5 mol/L sucrose solution in the same concentration. For example, where the total final AA concentration was equal to 0.1 M, each individual AA in that solution was at a concentration of 0.01 M (Paoli et al. 2014). From here-on, this AA mix is referred to as the “equimolar mix”. The second AA mix is referred to as the “pollen mix”. Here, the proportions of each AA were the same as that collected by *B. terrestris* foragers in Leonhardt and Blüthgen (2012). In this earlier study, the AA content of pollen retrieved by two *B. terrestris* colonies was assayed. The 10 AAs in our diets only represent a subset of the AAs in this pollen and so, we averaged the proportion of each AA of interest collected by the two colonies and then scaled these proportions such that they summed to 1 (Table S1).

Each of these two AA mixes (equimolar and pollen) was then used to construct diets that varied in their AA:C ratio. Seven AA:C ratios were created 0:1, 1:250, 1:100, 1:75, 1:50, 1:25 and 1:10 AA:C and ratios were calculated on a molar–molar basis (e.g., 1:10 = 0.05 M AAs, 0.5 M sucrose). As in Stabler et al. (2015) the sucrose concentration was 0.5 mol / L in all experimental diets, but the portion of AAs in each diet differed. It is therefore, important to highlight that the total nutrient composition of diets (AA + C) was not constant across dietary treatments.

Two tubes of each experimental diet were provided to each micro-colony, together with a tube containing demineralised water. The weight of the two vials was measured when food was provided, and then measured again 24 h later when tubes were replaced. Simultaneously, one control cage per experimental diet was established. These control cages were identical to experimental colonies but did not include bees and were used to calculate evaporative loss of each diet (i.e., mass change over 24 h) (Supplement Table S2). Daily food consumption was calculated as the mass change in experimental tubes each day, minus the average evaporative water loss over 24 h for the same experimental diet (i.e., the mean of seven evaporative values per diet). Consumption was then calculated per bee alive in the cage during the previous 24 h, and converted into a volume by dividing the daily consumption value by the density of each solution (1.06 as in Stabler et al. 2015). Total diet consumption is the sum of these daily values scaled per bee surviving in each microcolony over the feeding experiment. AA or C consumption was calculated as the volume of each diet consumed multiplied by the molarity of AA and C in that solution [i.e., volume (ml) × molarity = number of millimoles consumed]. Bees were fed daily over 7 days.

In bees fed the pollen AA mix, 15 micro-colonies were allocated to each of the AA:C ratios (15 micro-colonies × 5 individual bees × 7 diets = 525 bees), such that each trait of interest (body composition, survival, ovarian activation) could be assayed in all surviving members of each of five different micro-colonies fed each AA:C ratio. Traits were measured in different micro-colonies to ensure that members of each starting colony were assayed. Repeating this full experiment using the equimolar mix was not possible—starting colonies were too small—and so five micro-colonies were allocated to each of the AA:C ratios and the equimolar AA mix, all of which were used to assay ovarian activation at the end of the experiment (5 micro-colonies × 5 individual bees × 7 diets = 175 bees), which a priori we predicted was most likely to be affected by dietary AA mix. This experimental design is summarised in Table S3, and the number of bees assayed for each trait of interest is shown in Table S4.

### Experiment 1: testing the effects of nutrition on fitness traits

#### Measuring wing length

So that we could use body size as a covariate in analyses, the marginal cell length of each wing was measured (Supplement Fig. S1). To achieve this, the left forewing of each individual was removed and fixed to a microscope slid with transparent nail polish. Slides were photographed under a microscope (Objective at 1x, zoom at 0.8x, reflector at ‘BF’, total magnification at 0.4) and marginal cell length was measured using the image capture program Zen 3.0 pro by Carl Zeiss Microscopy GmbH. All marginal cells were measured independently by each of two experimenters to maximise measurement accuracy. If these values differed, wings were measured a third time. If the left wing was damaged, the right was used instead. The average of these two measurements was used as a proxy for overall bee size.

#### Abdomen composition

Dry mass is a measure of size, which correlates positively with multiple traits associated with fitness in bumble bees (Hagen and Dupont 2013). In *B. hypnorum*, dominant workers tend to have a larger fat body (Ayasse et al. 1995) and longevity under starvation conditions is positively correlated with fat body mass in *B. terrestris* (Manley et al. 2017). Accordingly, abdomen weight and fat body mass are both indicators of individual condition. Body composition was assayed in animals from each of five randomly selected micro-colonies of bees fed the pollen AA mix and each AA:C ratio (Sample sizes in Table S4). To measure abdomen dry and lipid mass, at the end of feeding experiments each bee was euthanized by freezing at − 80 °C. Individuals were removed from the freezer and their abdomens separated from the rest of their bodies. Abdomens were cut in half longitudinally, transferred to a glass vial of known weight and weighed immediately. Thereafter, vials were dried in a drying oven for 72 h at 70 °C. After 72 h, vials were re-weighed, before being put back in the oven for 24 h and re-weighed 24 h later to confirm that samples had reached their dry mass (first dry mass measure). 2 ml of Folch’s reagent (chloroform:methanol 2:1 v/v) was then added by a glass syringe to each vial before it was sealed. Folch’s reagent was removed and replaced every 24 h for 4 days. After four washes, the Folch’s reagent was removed and the vials were unsealed in a fume hood for 24 h to ensure full evaporation of the reagent. The drying process was repeated in the oven at 70 °C for 4 days. Vials were weighed and the difference between the first and second dry measurement is our measure of lipid content of each sample. The second dry mass is our lipid free dry mass measurement.

#### Survival

Survival was recorded daily in all experimental animals during the feeding experiments. Survival following food removal was then assayed in five micro-colonies fed each AA:C ratio, created using the pollen AA mixture (Sample sizes in Table S4)*.* 24 h after their last feed (noting that food was remaining in Eppendorf tubes when it was removed from the cage), each bee was transferred into a plastic vial (70 × 47 mm) without food or water. Survival of these bees was monitored every 30 min for 48 h and the time of death in minutes was recorded. Any bees surviving at the end of the 48 h observation period (*N* = 3) were recorded as being alive and their survival values censored in analyses. Surviving animals were then euthanized by freezing.

#### Ovarian activation

Ovarian activation was measured in 35 micro-colonies (5 fed each AA:C ratio), fed the pollen AA mixture and the equimolar AA mixture (Sample sizes in Table S4). All experimental animals were euthanized by freezing at – 80 ºC. To assay ovarian activation, bees were left to defrost briefly before they were dissected to remove the ovaries, which were then photographed using Zen 3.0 pro by Carl Zeiss Microscopy GmbH. The ovaries were classified as being fully activated, partially activated or inactive—Supplementary Fig. S2 shows examples of each activation level.

### Statistical analyses

All analyses were conducted in R version 4.0.3 (R Core Development Team 2017) and all plots created using this program. Statistical significance of effects assessed using the Anova function in the “car” package (Fox and Weisberg 2018), unless otherwise stated.

Lipid content and abdomen dry mass excluding lipid content were analysed in separate models. Lipid dry mass was square-root transformed, such that data residuals met the pre-requisites of parametric analyses, and analysed in a linear mixed model in the lme4 package (Bates et al. 2015) with wing length included as a covariate (scaled, continuous) and diet (scaled, continuous) as a main effect, with micro-colony ID number as a random term (35 levels). Because the carbohydrate content of experimental diets was constant but the portion of AAs in each diet varied, the AAs in each diet (in moles) was used a continuous explanatory variable to test for effects of dietary maniupulation. For abdomen dry mass (excluding lipids), because data were right-skewed and the model residuals non-normally distributed, models were fitted with family = inverse Gaussian (link = inverse) using a generalised linear mixed-effects model using the function “glmer” in the lme4 package (Bates et al. 2015). Explanatory variables and random effects were as described above for models analysing lipid content.

Survival following food removal was analysed using the “coxme” function in the survival package (Therneau 2020b) with wing length as a covariate and dietary AA content as a continuous explanatory variable, and micro-colony ID as a random effect. Hazard was calculated relative to bees fed the sucrose only diet. Survival during the experiment was analysed in the same way, but wing length was not included, because this trait was not measured in animals that died prior to the end of the experiment. In addition, because this trait was monitored in all experimental animals, this model included an interaction between AA mix (equimolar/pollen) and dietary AA content. The baseline hazard was calculated for the 0:1 AA:C ratio and the equimolar AA mix. In both cases, we used scaled Schoenfield residuals for a model without random terms using the function cox.zph (Therneau 2020a) to test the proportionality assumption of each model.

Ovarian activation was scored as a factor with three levels (inactive, partially activated, fully activated) accordingly, we analysed these data as a multinomial mixed model in *MCMCglmm* (Hadfield 2010). We fitted variances but no covariances for our fixed effects, and the random effects of cage. Initially, models included wing length as a covariate; however, this led to a high level of autocorrelation. Given this, and because wing length should not affect model outcomes—bees were randomly allocated to diets and so while wing length may correlate with ovarian activation it should not drive any trends related to diet—this parameter was excluded from analyses. We fitted a series of models that varied in complexity from null (just “trait” as a predictor) to full (AA:C ratio—continuous, AA mix—factor, interaction between AA:C and AA mix) and compared these models with the DIC function (Supplementary Table S5). Each model was run for 275,000 iterations with a thinning interval of 100 and a burn-in of 75,000 with parameters suggested by Hadfield for multinomial models (Hadfield 2021, p 97). This resulted in 2000 samples from the posterior with low autocorrelation between successive samples.

### Experiment 2: characterising how bees regulate their nutrient intake when constrained to a single nutritionally imbalanced diet

Dietary intake was monitored in each experimental micro-colony and these data used to test how bees regulate their intake when constrained to a single diet. First, the total volume of each diet consumed per bee was analysed using a generalised linear model with AA mix (factor, 2 levels) and dietary AA content (continuous) as explanatory variables. Micro-colony ID was not included as a random effect, because there was only one observation per micro-colony. Data were positively skewed and so the family inverse Gaussian was used.

**Fig. 1:**
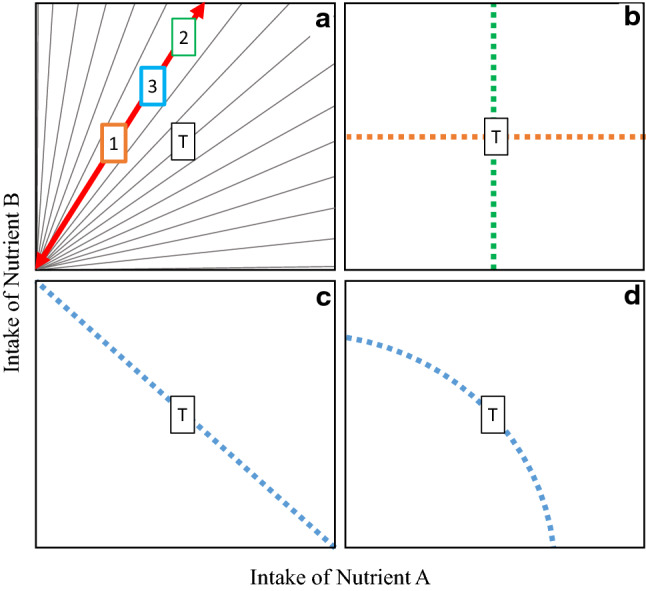
Rules of compromise. **a** Grey lines—each nutrient rail represents a single diet varying in its ratio of nutrient A to nutrient B. Aligned along the red arrow are three possible solutions to the challenge of being unable to consume the intake target (T). **b** If individuals are fed different diets and consistently chose strategy 1 in (**a**), the intake array will be as shown in the orange line. If all individuals choose strategy 2, then the intake array will be as in the green line. Both of these strategies represent the no-interaction rule of compromise. **C** If all individuals in an experiment choose option 3 in (**a**), then the intake array may take this form—the equal distance rule. **d** Alternatively, individuals that adopt strategy 3 in panel A, may be adopting the closest distance rule. This schematic is based on figures from Raubenheimer and Simpson 1999

To test how bumble bees regulated their intake of AAs and carbohydrate when constrained to a single nutritionally imbalanced diet we constructed intake arrays: lines that connect average intake of each of the experimental diets. Intake arrays provide a visual representation of how animals trade-off the costs of over- versus under-consuming nutritionally imbalanced foods when constrained to a single diet (Simpson et al. 2004). Example intake arrays are shown in Fig. 1, in a schematic based on figures in Raubenheimer and Simpson 1999. There is an optimal ratio of nutrients A and B for fitness (intake target ratio—illustrated by a “T” in each figure). Rather than being free to regulate their intake of nutrients A and B, individuals are fed one of several nutritionally imbalanced diets (represented by grey lines in Fig. 1a). If an organism is fed the nutrient ratio highlighted in red in Fig. 1a, the organism can 1) eat the right amount of nutrient B, but under-ingest nutrient A, 2) eat the right amount of nutrient A, but over-ingest nutrient B or 3) compromise and eat a little too much of nutrient B, and slightly too little of nutrient A. Intake arrays measure how different animals resolve this trade-off on a range of imbalanced diets and so reveal “global rules of compromise”. In Fig. 1b, animals have regulated their intake of nutrient A (green dashed line) or B (orange dashed line), without attempting to rectify shortfalls or excess consumption of the other nutrient. This is called the no interaction rule. In Fig. 1c, animals regulate their intake such that deficits in one nutrient are exactly balanced by surpluses in the other—the equal distance rule—while in Fig. 1d, animals appear to be feeding to the ratio of A:B which is closest to their target, given the rail they are confined do—the closest distance rule.

We created intake arrays for bumble bees fed the equimolar and the pollen AA mixes to test whether dietary AA blend altered global rules of compromise. To analyse these arrays, we first estimated the slope for average total intake of AAs and carbohydrate for each 7 diets. A linear slope of -1 would be indicative of the equal distance rule and suggest that individuals give an equal priority to regulating both AAs and carbohydrate. If the slope of the intake array is significantly <  − 1, individuals are regulating their intake of carbohydrate more strongly than their intake of AA, whereas a slope that is significantly >  − 1 indicates the reverse is true and organisms regulate their intake of AA more strongly than carbohydrate (i.e., characterised by the “no interaction rule” of compromise—Fig. 1). To compare the slope of the array with hypothetical slopes of 0 and -1, we created a linear model with mean AA intake per bee as an explanatory variable, and mean carbohydrate intake per bee as the response variable. The slope of the intake array for each AA mix (*β*_a_) was tested against a hypothetical slope (*β*_h_) of − 1 and 0 using a *t*‐test, where (*β*_a_ − *β*_h_) / SEβa approximates a *t*‐distribution with *n* − 2 degrees of freedom.

Because there was some curvature in the shape of the arrays, we used the function “PFunc” (Kilmer et al. 2017) to characterise the shape of each array. This tool was developed in behavioural / evolutionary ecology to analyse female mate preference functions, but generally provides a way of analysing function valued traits. For each AA blend, we characterised (a) the value on the *x* axis (the AA molarity of each experimental diet) that elicits the greatest response (the sum of carbohydrate consumption per bee over the 7-day experiment) (peak), (b) the maximum elevation of the function on the *y*‐axis at the peak point (height), (c) the width of the function at a given elevation relative to the height of the peak (tolerance) and (d) the degree to which carbohydrate intake falls away from peak intake values (strength). This is the first time, to our knowledge, that intake arrays have been analysed in this way. Our aim is to minimise subjectivity when comparing two non-linear arrays.

## Results

### Experiment 1: testing the effects of nutrition on fitness traits

#### Abdomen body composition

**Fig. 2:**
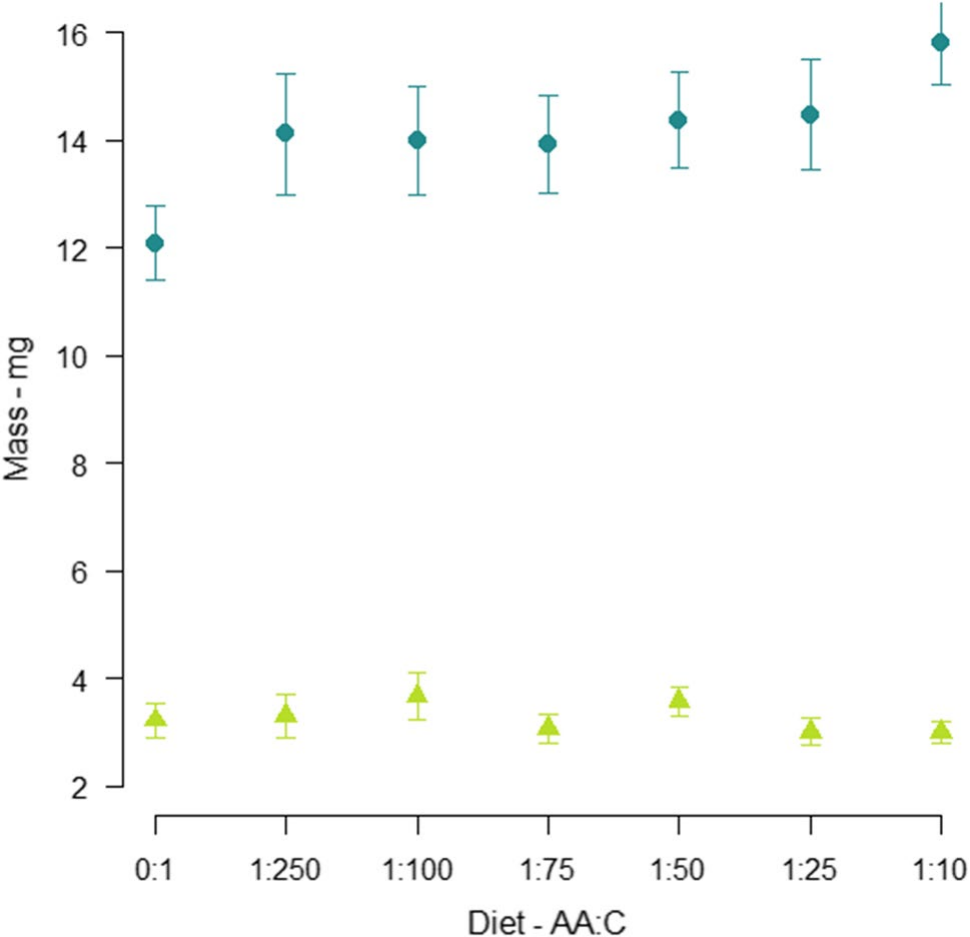
Abdomen composition. Mean abdomen dry mass (± SE) excluding lipids (turquoise circles) and lipid mass (yellow triangles) in bees fed each of the AA:C ratios

There was a positive relationship between individual bee lipid content and its wing marginal cell length (Supplement Fig. S3, *X*_2_ = 50.37, *df* = 1, *P* ≤ 0.001) but lipid content was unaffected by diet (*X*_2_ = 0.099, *df* = 1, *P* = 0.753) (Fig. 2). Dry mass of each bee (excluding lipids) was affected by wing length (Supplement Fig. S4, *X*_2_ = 255.86, *df* = 1, *P* ≤ 0.001) and positively associated with dietary AA content (*X*_2_ = 14.91, *df* = 1, *P* ≤ 0.001) (Fig. 2).

#### Survival

**Fig. 3:**
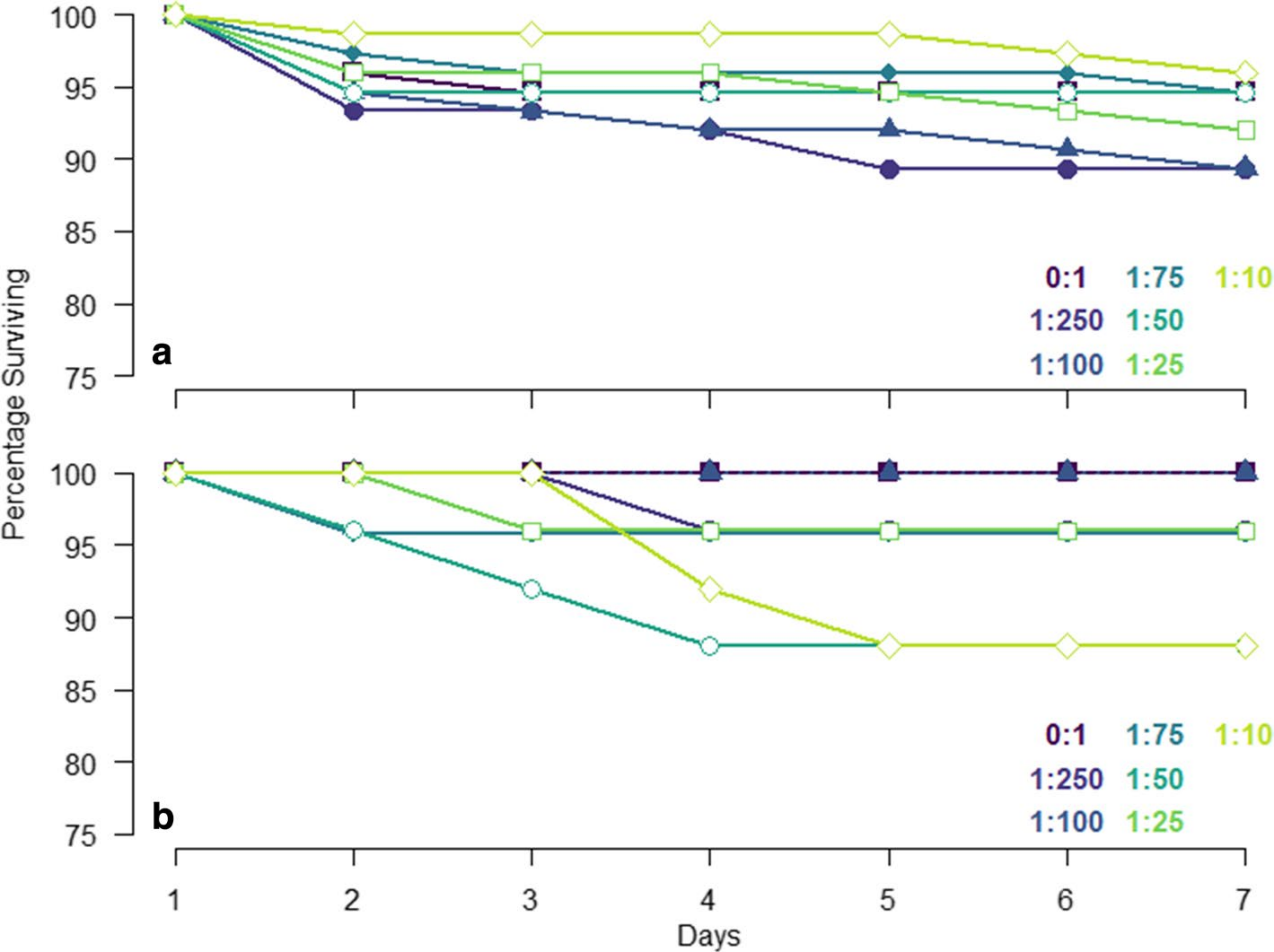
Survival during the 7-day experiment. Percentage of animals surviving over the 7-day feeding experiment fed the pollen AA mix (**a**) or the equimolar AA mix (**b**). Filled square—0:1 AA:C, filled circle—1:250 AA:C, filled triangle—1:100 AA:C, filled diamond—1:75 AA:C, open circle—1:50 AA:C, open square—1:25 AA:C; open diamond—1:10 AA:C

**Fig. 4:**
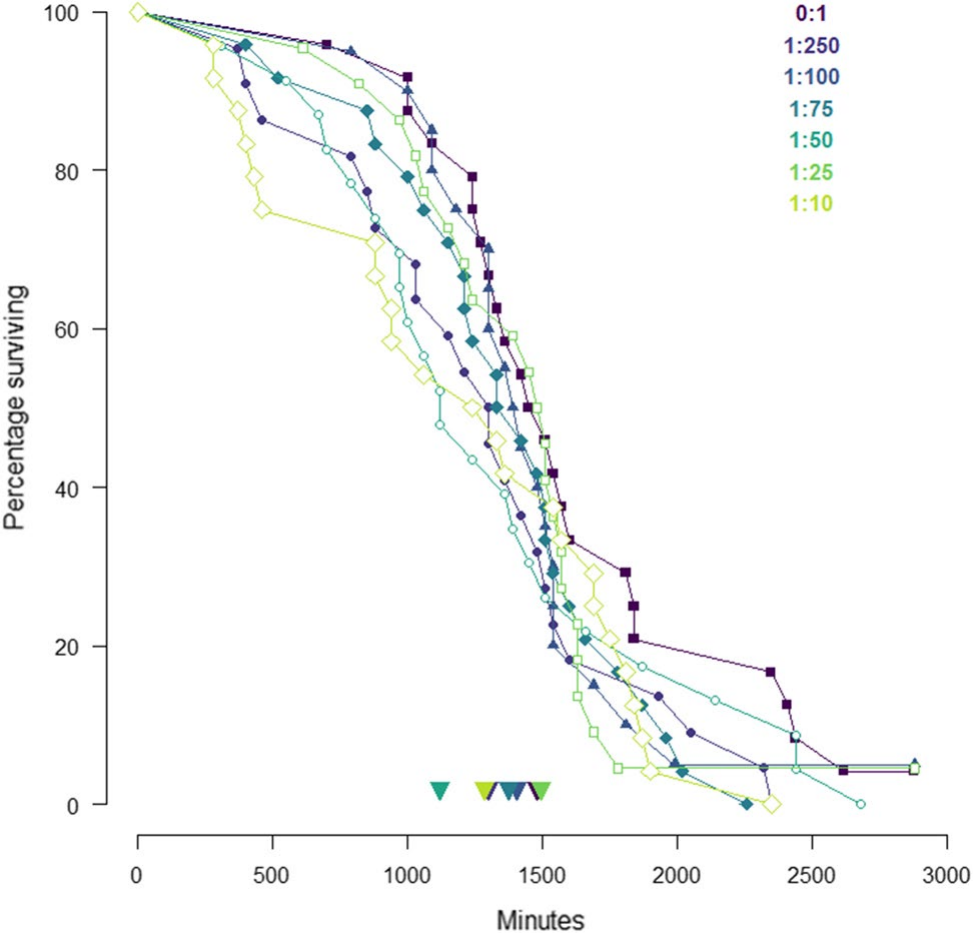
Percentage of bees fed each experimental diet surviving in minutes after their food was removed. Filled square—0:1 AA:C, filled circle—1:250 AA:C, filled triangle—1:100 AA:C, filled diamond—1:75 AA:C, open circle—1:50 AA:C, open square—1:25 AA:C; open diamond1:10 AA:C. Median lifespan for each diet is shown by colour-coded arrows aligned along the *x*-axis

During the 7-day feeding experiment survival was high. The main effects of AA mix (*X*_2_ = 0.26, *df* = 1, *P* = 0.61) and AA:C ratio (*X*_2_ = 0.03, *df* = 1, *P* = 0.87) were non-significant, but, there was a significant interaction between AA mix and AA:C ratio (*X*_2_ = 4.02, *df* = 1, *P* = 0.045) (Fig. 3). Mortality risk following food removal was affected by wing length (*X*_2_ = 4.58, *df* = 1, *P* = 0.032), with larger bees having a lower likelihood of dying, but not by AA:C ratio (*X*_2_ = 0.39, *df* = 1, *P* = 0.535) (Fig. 4).

#### Ovarian activation

**Fig. 5:**
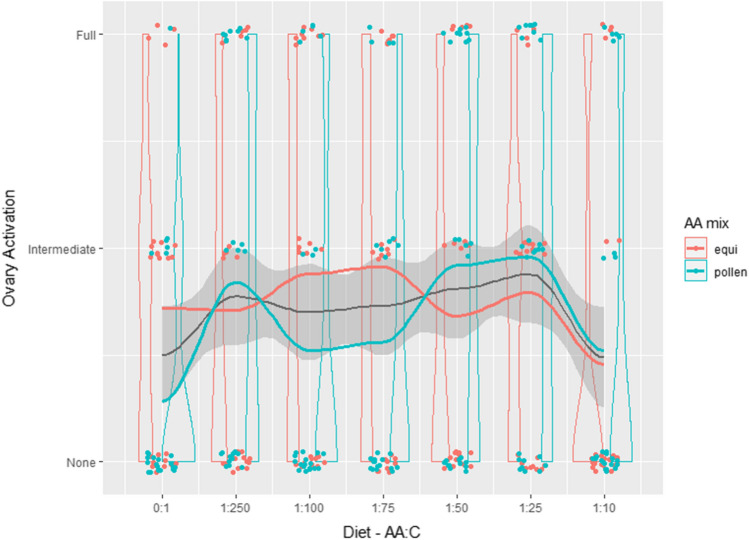
Ovarian activation. Ovarian activation in bees fed pollen AA mixture (turquoise) and equimolar AA mixture (orange) and each AA:C ratio. Lines show smoothed curves for the two AA mixes individually (turquoise and orange), and for the pooled data (grey line with shaded area representing the standard error). Figure created using ggplot (Wickham 2016)

The multinomial model with the lowest DIC value (Supplement Table S5) was the null model showing that ovarian activation was independent of AA:C ratio and dietary AA mix (Fig. 5).

### Experiment 2. Characterising how bees regulate their nutrient intake when constrained to a single nutritionally imbalanced diet

**Fig. 6:**
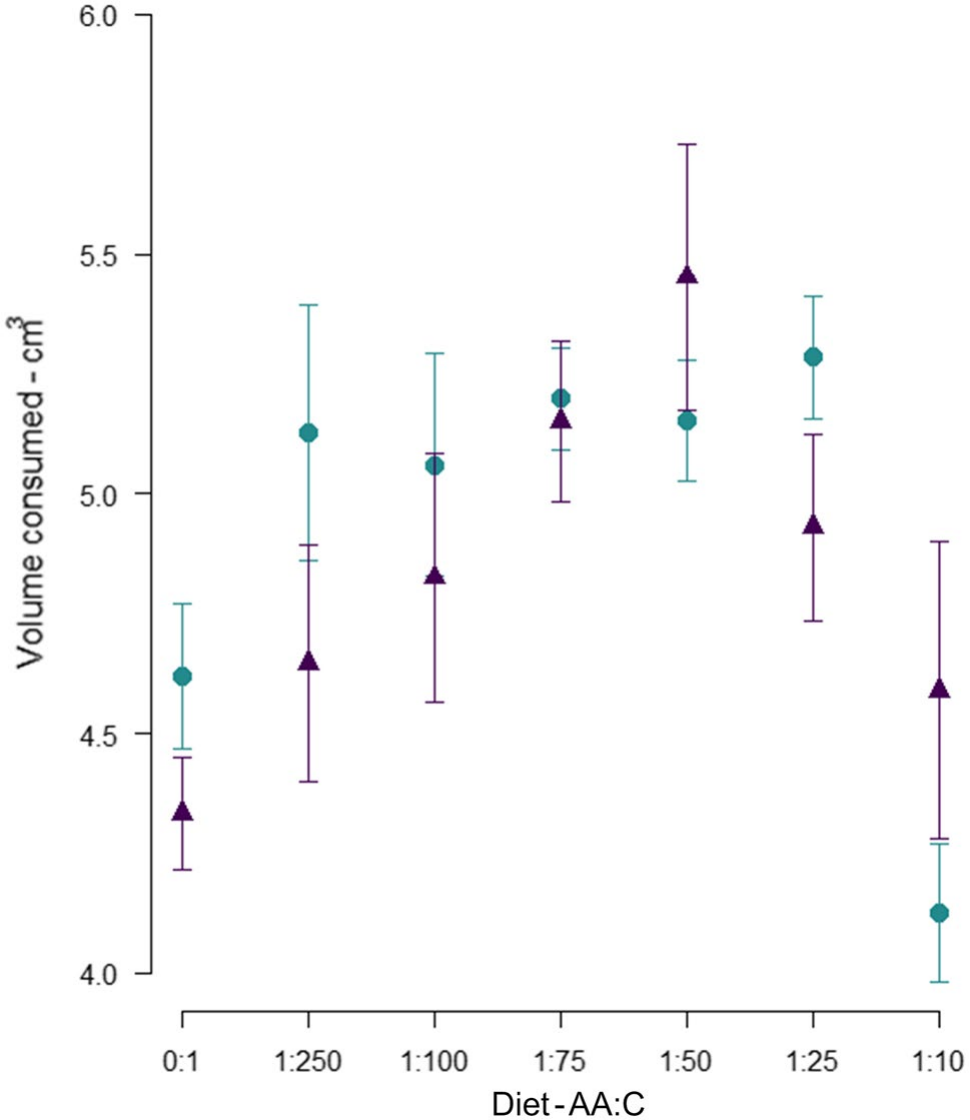
Volume of each experimental diet consumed. Total volume of each experimental diet consumed on average per bee across the entire experiment in individuals fed the pollen AA mixture (turquoise circles) and an equimolar AA mixture (purple triangles) (mean ± SE)

Total nutrient intake per bee across the experiment was affected by AA:C ratio (*X*_2_ = 14.26 *df* = 1, *P* =  < 0.001), but not by AA mix (*X*_2_ = 0.17, *df* = 1, *P* = 0.675). There was, however, a weakly significant interaction between AA mix and AA:C ratio (*X*_2_ = 3.89, *df* = 1, *P* = 0.049). To better understand the effects of AA:C ratio on total intake (as these effects appeared non-linear) models were re-run with AA:C ratio coded as a factor and post hoc tests performed using “emmeans” (Lenth 2016). In this second analyses, the interaction between AA mix and AA:C ratio became non-significant. Post-hoc tests of this model showed that the effects of diet AA:C ratio were driven by consumption of the 1:10 AA:C diets being significantly lower than intake of 1:75, 1:50, 1:25 AA:C and consumption of the 0:1 AA:C diet being significantly lower than diets 1:75 and 1:50 AA:C. Accordingly, intake of diets of intermediate AA:C ratio was greater than intake of either very high, or very low, AA:C ratios (Fig. 6).

When analysing mean intake of AAs and carbohydrate, the slope of the relationship for bees fed the pollen mixture was tilted towards the horizontal axis, with a slope of − 1.68 (± 1.05 SE). However, the large error term associated with this slope meant that it did not differ significantly from a hypothetical slope of − 1 (*T* = − 0.685, *df* = 5, *P* = 0.542) or 0 (*T* = − 1.609, *df* = 5, *P* = 0.169). The slope of the mean intake in bees fed the equimolar AA mix was also negative − 0.127 ± 1.05, and did not differ from a hypothetical slope of -1 (indicative of the equal distance rule of compromise) (*T* = 0.835, *df* = 5, *P* = 0.442) or 0 (indicative of the no interaction rule with strong prioritisation of carbohydrate intake) (*T* = − 0.122, *df* = 5, *P* = 0.908). The large errors in these slopes hint at curvature in the shape of the relationship between mean AA and mean carbohydrate intake over the course of the experiment.

**Fig. 7:**
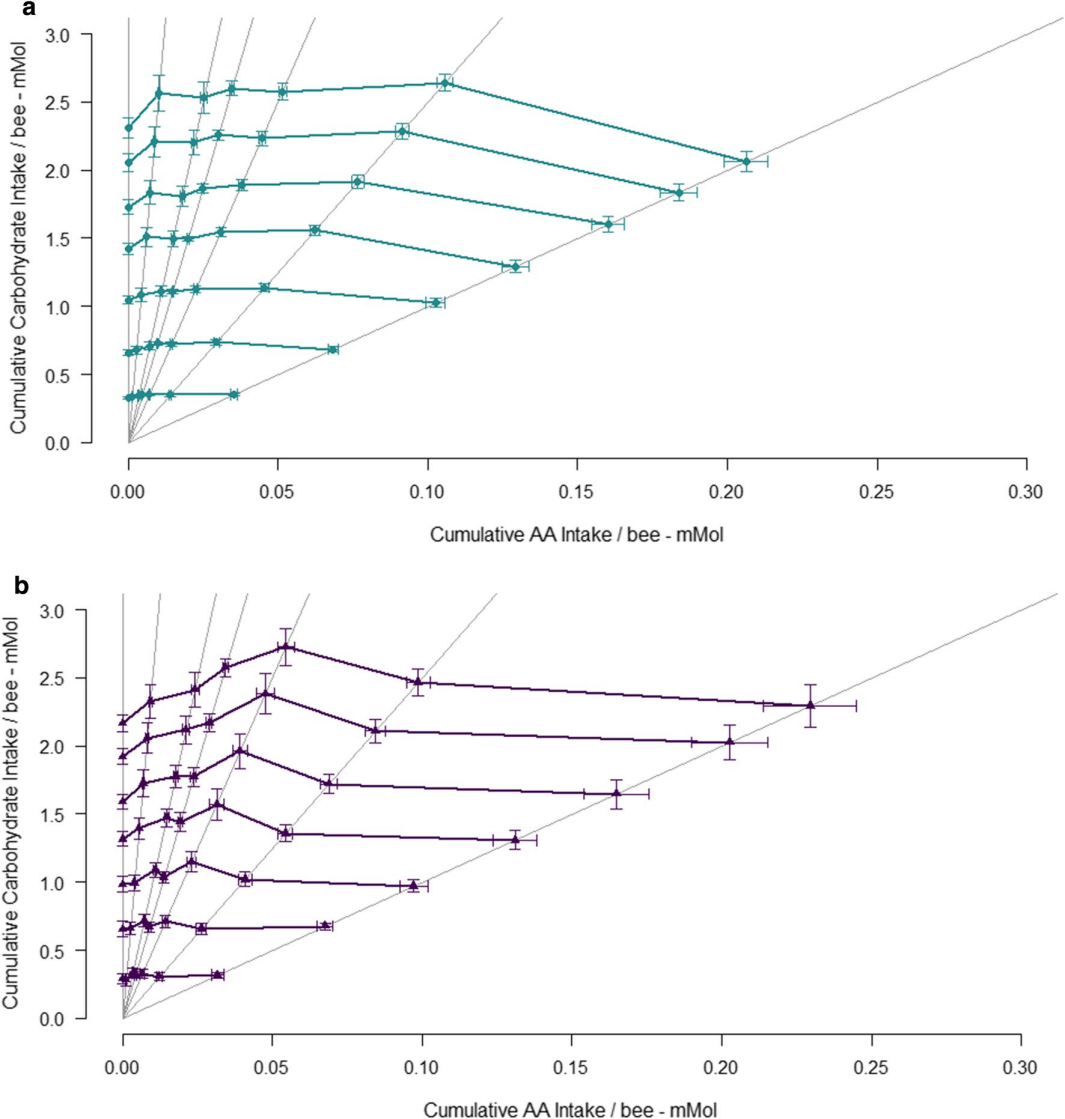
Intake arrays. Mean cumulative intake of AAs and carbohydrate (± SE) consumed per bee by individuals fed pollen AA mixture (**a**) and an equimolar AA mixture (**b**). Each of the 7-day intake values is shown

Visual inspection of the intake arrays (Fig. 7) suggests that there is slight curvature and that perhaps bees may adopt a closest distance compromise rule—particularly in bees fed the equimolar mix (see Fig. 1d). Using the package PFunc to characterise the shape of these arrays (created for total intake over 7 days) showed that the peak in the array was at 0.019 mmol AA consumption for the pollen AA mix, but 0.023 mmol AA for the equimolar mix, i.e., the peak in the array was shifted towards slightly higher AA content in equimolar diets. The height—indicative of average carbohydrate intake across experimental diets—was similar for the pollen and equimolar AA mixes (pollen: 2.65; equimolar: 2.59), but the strength (difference in consumption between the diets that are consumed in large versus low amounts) was slightly higher for the equimolar mix (pollen: 0.328; equimolar: 0.349), suggesting slightly greater curvature in the shape of the array.

## Discussion

We aimed to test how the AA:C ratio that bumble bees (*Bombus terrestris*) consumed affected multiple traits that indicate overall bee condition and to ask how ovarian activation, survival during feeding and food intake varied in bees fed the same AA:C ratios, but where the AA complement of the food differed. We found that abdomen dry mass (excluding lipids) increased as diets became richer in AAs, but AA:C ratio did not significantly affect survival following food removal or ovarian activation. AA blend (i.e., equimolar versus pollen) did not affect overall ovarian activation or food consumption. However, the interaction between AA blend and AA:C ratio was significant for survival during the experiment and there were signs of a possible interaction affecting total food intake during the experiment. Crucially, we do not find overt support for a dietary mediated trade-off between the fitness traits assayed here. While more work is needed, we suggest that the relationship between AAs, bee fitness and food consumption is complex and that delving into the AA complement of experimental diets may be a key step in better understanding bee nutritional ecology.

We find that the AA:C ratio in experimental diets had a pronounced effect on bee size: abdomen dry mass (excluding lipids) increased as the AA content of experimental diets rose. This result is not just due to variation in experimental diet remaining in bee guts—which were not dissected prior to mass measurement—because intake of the 1:10 AA:C diet was the lowest of any experimental diet. We speculate that differences reflect greater protein accumulation in bees fed higher AA:C ratios. Comparison of the protein content of small and large bumble bee workers shows that muscular proteins were more abundant in the abdomens of large workers (Wolschin et al. 2012), reflecting perhaps that larger bees are more likely to forage outside the nest than their smaller colony mates who tend to focus on in-nest tasks (Yerushalmi et al. 2006).

Whatever the underlying mechanism, the impact on fitness is clear—large size is positively correlated with several fitness traits in bees. A comparative analysis of 62 bee species from 6 families, where intertegular span was used as a proxy for body mass showed that larger bees forage further (Greenleaf et al. 2007). Similarly, heavier bumble bees forage for nectar at a higher rate than small colony members, and thus contribute disproportionately to nectar retrieval (Spaethe and Weidenmüller 2002). In *B. impatiens* larger bees (measured as head width and radial wing cell length) learn more quickly than smaller bees (Worden et al. 2005) and brain size is generally bigger in heavier individuals, perhaps explaining the improved foraging efficiency of larger colony members (Mares et al. 2005). Brain size is not fixed at adult development in bumble bees but rises with age and is affected by experience (Jones et al. 2013), meaning that changes in mass due to adult nutrition may still be positive indicators of brain size. A large size may also help buffer the impacts of habitat fragmentation—as habitats become increasingly fragmented there is selection for large body size if this facilitates dispersal between patchy habitats (Merckx et al. 2018). In keeping with this prediction, *B. terrestris* tend to be larger in fragmented urban habitats (Theodorou et al. 2021). Being large is not without cost: larger bees are more vulnerable to overheating when the ambient air temperatures are high for example (Goulson 2010). However, at colony level producing some large workers clearly has fitness benefits. These data show that high AA consumption may be one means of promoting the development of larger bodied individuals after bees emerge as adults.

Dietary AA:C ratio was not significantly associated with survival following food removal, but there was a significant interaction between AA mix and AA:C ratio affecting survival during the feeding experiment. This interaction term likely reflects that in bees fed the pollen mix, there was no clear pattern in mortality with respect to dietary AA content, but on the equimolar mix only two bees died in diets containing diets with AA content equal to, or less than, 1:75 AA:C, while 7 bees died containing diets that were richer in AAs. This is in keeping with past work. Ruedenauer et al. (2020) found no effects on bumble bee survival of enriching pollens with AAs provided in the average ratios they appear in pollen. However, Stabler et al. (2015) found that bumble bees fed diets with high amounts of AAs provided in equimolar ratios did experience reduced survival. In combination with the significant interaction term observed here, a picture is emerging where the effects of total dietary AA content appear to be contingent on the ratio of individual AAs relative to one another in experimental diets. This has important implications for dietary manipulation work in bees and highlights the importance of optimising, and standardising, experimental diets.

The apparent costs of high AA intake observed in bees fed an equimolar AA mix seen here are modest—indicated only by an interaction term between AA mix and AA:C ratio. These effects are less pronounced than observed in past studies (Stabler et al. 2015). One possible explanation for this difference is the social environment—in the current experiment bees were housed in micro-colonies rather than individually (as in Stabler et al. 2015). Housing bees individually provides greater resolution in terms of quantifying nutrient intake and characterising strategies of nutrient regulation, but behaviours and physiology differ between individually housed bumble bees and bees in queenless micro-colonies. For example, in our main and preliminary experiments we observed bees building brood cells and fighting, and bees in micro-colonies experience ovarian activation. These behaviours and physiological changes do not occur in individually housed bees—egg laying is stimulated by social interactions for example, although suppressed by the presence of a queen (Duchateau and Velthuis 1989) and aggression precedes ovarian activation in queenless *B. terrestris* microcolonies (Amsalem and Hefetz 2010). Accordingly, one (speculative) explanation for our results is that behaviours and physiological changes associated with being in queenless micro-colonies may somehow buffer against the cost of high AA intake. Exploring the impact of social environment on bee nutrition might be an interesting avenue of future investigation. A simpler scenario is that, if survival is generally higher in bees housed in micro-colonies than in individually housed bees it may take a longer observation period or a larger sample size for significant differences driven by diet to emerge in bees housed in micro-colonies. This seems feasible given that in honey bees the density of bees in the social environment is an important predictor of survival in caged feeding experiments—when too many or too few bees are housed together there are reductions in survival (Bosua et al. 2018).

Ovarian activation was not significantly affected by AA:C ratio or by AA blend in experimental diets. Qualitatively, however, both very low (e.g., 0:1 AA:C) and very high AA:C (1:10 AA:C) ratios were associated with low ovarian activation, and ovarian activation was greatest on intermediate AA:C ratios (pollen AA mix—1:25 AA:C, equimolar AA mix—1:75 AA:C). Accordingly, we suggest that the absence of a significant result here may reflect low statistical power and that these results do not warrant rejecting a role for AA:C ratio in influencing bumble bee ovarian activation quite yet. This is particularly true because in a broad range of insect species, female fecundity is greatest in individuals that consume moderate to high ratios of protein to carbohydrate (Archer et al. 2015; Fanson et al. 2009; Harrison et al. 2014; Lee et al. 2008; Maklakov et al. 2008; Rapkin et al. 2017).

A second aim of the current work was to explore how AA mix itself affected bee nutrient regulation when constrained to a single nutritionally imbalanced food. There was a borderline significant interaction effect between AA mix and AA:C ratio affecting total food consumption. However, the effect size of this term was small and this significant interaction was lost when AA:C ratio was coded in analyses as a factor—thus we interpret this interaction term cautiously, and more work is needed to validate if this represents a biologically meaningful result. However, the shape of intake arrays appeared to differ slightly between each AA mix. In both arrays there was slight curvature, particularly in the intake array created using the equimolar AA mix. Curvature in intake arrays suggests that individuals may be adopting the closest distance rule of compromise, where individuals feed to the ratio of nutrients that on each nutrient rail is geometrically closest to their intake target ratio (the ratio animals would consume if able to regulate their intake from across the entire nutrient landscape) (Raubenheimer and Simpson 1999). This strategy reduces the costs of undereating one nutrient and overeating the other and so is relatively common in specialist insects. However, curvature in the shape of the arrays was not pronounced, and for the pollen AA mix over much the array carbohydrate intake was relatively constant, while AA intake varied. Clearly more work is needed to better understand how AA blend influences nutrient regulation, but we believe that subtle variation in the shape of these arrays suggest that the ratio of AAs relative to one another, as well as relative to carbohydrate, may influence nutrient intake strategies.

These data show that both AAs and AA:C ratio may affect bee dietary intake and fitness; however, these effects are complex. While many existing studies suggest that low AA:C ratios are good for bee survival, our result is in keeping with a recent study showing that increasing the AA content of diets need not reduce lifespan (Ruedenauer et al. 2020). The significant interaction we see between AA:C mix and AA ratio affecting survival, particularly when interpreted alongside existing data using diverse AA mixes in experimental diets, suggests that whether high overall AA intake reduces survival depends on the AA blend in diets. Furthermore, there were signals of a possible interaction between AA:C ratio and AA mix affecting total food consumption and subtle differences in the shape of the intake arrays in bees fed each AA mix. More work is needed to determine if these borderline results are ecologically meaningful, but in the interim, these results flag that controlling the AA blend of experimental diets in bee dietary manipulation work may be important—if effects on phenotype depend on the AA mix in experimental diets, then conclusions about dietary optima for bees will vary between labs, depending on dietary AA composition. Evidently, while bee diets are relatively simple—based on pollen and nectar—their nutritional ecology is complex and more work is needed to fully understand how nutrition affects phenotype in these important pollinators.

## Supplementary Information

Below is the link to the electronic supplementary material.

Supplementary file1 (DOCX 2348 kb)

## Data Availability

All data will be archived on Dryad on manuscript acceptance.

## References

[CR1] Abrol DP, Kapil RP (1991). Foraging strategies of honeybees and solitary bees as determined by nectar sugar components. Proc Indian Natl Acad Sci.

[CR2] Altaye SZ, Pirk CWW, Crewe RM, Nicolson SW (2010). Convergence of carbohydrate-biased intake targets in caged worker honeybees fed different protein sources. J Exp Biol.

[CR3] Amsalem E, Hefetz A (2010). The appeasement effect of sterility signaling in dominance contests among *Bombus terrestris* workers. Behav Ecol Sociobiol.

[CR4] Archer CR, Köhler A, Pirk CWW, Oosthuizen V, Apostolides Z, Nicolson SW (2014). Antioxidant supplementation can reduce the survival costs of excess amino acid intake in honeybees. J Insect Physiol.

[CR5] Archer CR, Pirk CWW, Wright GA, Nicolson SW (2014). Nutrition affects survival in African honeybees (*Apis mellifera scutellata*) exposed to interacting stressors. Funct Ecol.

[CR6] Archer CR, Hempenstall S, Royle NJ, Selman C, Willis S, Rapkin J, Blount JD, Hunt J (2015). Testing the effects of dl-alpha-tocopherol supplementation on oxidative damage, total antioxidant protection and the sex-specific responses of reproductive effort and lifespan to dietary manipulation in Australian field crickets (*Teleogryllus commodus*). Antioxidants.

[CR8] Ayasse M, Marlovits T, Tengö J, Taghizadeh T, Francke W (1995). Are there pheromonal dominance signals in the bumblebee *Bombus hypnorum* L (Hymenoptera, Apidae)?. Apidologie.

[CR9] Bates D, Mächler M, Bolker B, Walker S (2015). Fitting linear mixed-effects models using lme4. J Stat Soft.

[CR10] Bosua HJ, Pirk CWW, Archer CR, Nicolson SW (2018). Comparing natural and laboratory conditions: effects of cage volume and honeybee density on survival and nutrient intake. Apidologie.

[CR11] Cameron SA, Sadd BM (2020). Global trends in bumble bee health. Annu Rev Entomol.

[CR12] de Groot AP (1952). Amino acid requirements for growth of the honeybee (*Apis mellifica L*.). Experientia.

[CR13] Duchateau MJ, Velthuis HHW (1989). Ovarian development and egg laying in workers of *Bombus terrestris*. Entomol Exp Appl.

[CR14] Fanson BG, Weldon CW, Pérez-Staples D, Simpson SJ, Taylor PW (2009). Nutrients, not caloric restriction, extend lifespan in Queensland fruit flies (*Bactrocera tryoni*). Aging Cell.

[CR15] Fox J, Weisberg S (2018). An R companion to applied regression.

[CR16] Goulson D (2010). Bumblebees: behaviour, ecology, and conservation.

[CR17] Goulson D, Nicholls E, Botías C, Rotheray EL (2015). Bee declines driven by combined stress from parasites, pesticides, and lack of flowers. Science.

[CR18] Greenleaf SS, Williams NM, Winfree R, Kremen C (2007). Bee foraging ranges and their relationship to body size. Oecologia.

[CR19] Grund-Mueller N, Ruedenauer FA, Spaethe J, Leonhardt SD (2020). Adding amino acids to a sucrose diet is not sufficient to support longevity of adult bumble bees. Insects.

[CR20] Hadfield JD (2010). MCMC methods for multi-response generalized linear mixed models: the MCMCglmm R Package. J Stat Soft.

[CR21] Hadfield JD (2021) MCMC Course Notes. https://cran.r-project.org/web/packages/MCMCglmm/vignettes/CourseNotes.pdf. Accessed 26 Sept 2021

[CR22] Hagen M, Dupont YL (2013). Inter-tegular span and head width as estimators of fresh and dry body mass in bumblebees (*Bombus* spp.). Insectes Soc.

[CR23] Harrison SJ, Raubenheimer D, Simpson SJ, Godin J-GJ, Bertram SM (2014). Towards a synthesis of frameworks in nutritional ecology: interacting effects of protein, carbohydrate and phosphorus on field cricket fitness. Proc R Soc B Biol Sci.

[CR25] Jensen K, Mcclure C, Priest NK, Hunt J (2015). Sex-specific effects of protein and carbohydrate intake on reproduction but not lifespan in *Drosophila melanogaster*. Aging Cell.

[CR26] Jones BM, Leonard AS, Papaj DR, Gronenberg W (2013). Plasticity of the worker bumblebee brain in relation to age and rearing environment. Brain Behav Evol.

[CR27] Kilmer JT, Fowler-Finn KD, Gray DA, Höbel G, Rebar D, Reichert MS, Rodríguez RL (2017). Describing mate preference functions and other function-valued traits. J Evol Biol.

[CR28] Lee KP, Simpson SJ, Clissold FJ, Brooks R, Ballard JWO, Taylor PW, Soran N, Raubenheimer D (2008). Lifespan and reproduction in *Drosophila*: new insights from nutritional geometry. Proc Natl Acad Sci.

[CR29] Lenth R (2016). Least-squares means: the R Package, "lsmeans". J Stat Soft.

[CR30] Leonhardt SD, Blüthgen N (2012). The same, but different: pollen foraging in honeybee and bumblebee colonies. Apidologie.

[CR31] Madliger CL, Love OP, Hultine KR, Cooke SJ (2018). The conservation physiology toolbox: status and opportunities. Conserv. Physiol..

[CR32] Maklakov AA, Simpson SJ, Zajitschek F, Hall MD, Dessmann J, Clissold F, Raubenheimer D, Bonduriansky R, Brooks RC (2008). Sex-specific fitness effects of nutrient intake on reproduction and lifespan. Curr Biol.

[CR33] Manley R, Boots M, Wilfert L (2017). Condition-dependent virulence of slow bee paralysis virus in *Bombus terrestris*: are the impacts of honeybee viruses in wild pollinators underestimated?. Oecologia.

[CR34] Mares S, Ash L, Gronenberg W (2005). Brain allometry in bumblebee and honey bee workers. Brain Behav Evol.

[CR35] Merckx T, Souffreau C, Kaiser A, Baardsen LF, Backeljau T, Bonte D, Brans KI, Cours M, Dahirel M, Debortoli N (2018). Body-size shifts in aquatic and terrestrial urban communities. Nature.

[CR36] Moatt JP, Savola E, Regan JC, Nussey DH, Walling CA (2020). Lifespan extension via dietary restriction: time to reconsider the evolutionary mechanisms?. BioEssays.

[CR37] Nakagawa S, Lagisz M, Hector KL, Spencer HG (2012). Comparative and meta-analytic insights into life extension via dietary restriction. Aging Cell.

[CR38] Nicolson SW (2011). Bee food: the chemistry and nutritional value of nectar, pollen and mixtures of the two. Afr Zool.

[CR39] Paoli PP, Donley D, Stabler D, Saseendranath A, Nicolson SW, Simpson SJ, Wright GA (2014). Nutritional balance of essential amino acids and carbohydrates of the adult worker honeybee depends on age. Amino Acids.

[CR40] Piper MD, Soultoukis GA, Blanc E, Mesaros A, Herbert SL, Juricic P, He X, Atanassov I, Salmonowicz H, Yang M (2017). Matching dietary amino acid balance to the in silico-translated exome optimizes growth and reproduction without cost to lifespan. Cell Metab.

[CR41] R Core Development Team (2017) A language and environment for statistical computing. R Foundation for Statistical Computing, Vienna, Austria. URL http://www.R-project.org/.

[CR42] Rapkin J, Archer CR, Grant CE, Jensen K, House CM, Wilson AJ, Hunt J (2017). Little evidence for intralocus sexual conflict over the optimal intake of nutrients for life span and reproduction in the black field cricket *Teleogryllus commodus*. Evolution.

[CR43] Raubenheimer D, Simpson SJ (1999) Integrating nutrition: a geometrical approach. In: Proc. 10th Int. Symp. Insect-Plant Relat. Springer, pp 67–82. 10.1007/978-94-017-1890-5_8

[CR44] Roulston TH, Cane JH (2000). Pollen nutritional content and digestibility for animals. Plant Syst Evol.

[CR45] Ruedenauer FA, Leonhardt SD, Lunau K, Spaethe J (2019). Bumblebees are able to perceive amino acids via chemotactile antennal stimulation. J Comp Physiol A.

[CR46] Ruedenauer FA, Raubenheimer D, Kessner-Beierlein D, Grund-Mueller N, Noack L, Spaethe J, Leonhardt SD (2020). Best be (e) on low fat: Linking nutrient perception, regulation and fitness. Ecol Lett.

[CR47] Simpson SJ, Raubenheimer D (2012). The nature of nutrition: a unifying framework from animal adaptation to human obesity.

[CR48] Simpson SJ, Sibly RM, Lee KP, Behmer ST, Raubenheimer D (2004). Optimal foraging when regulating intake of multiple nutrients. Anim Behav.

[CR49] Spaethe J, Weidenmüller A (2002). Size variation and foraging rate in bumblebees (*Bombus terrestris*). Insectes Soc.

[CR50] Stabler D, Paoli PP, Nicolson SW, Wright GA (2015). Nutrient balancing of the adult worker bumblebee (*Bombus terrestris*) depends on its dietary source of essential amino acids. J Exp Biol.

[CR51] Theodorou P, Baltz LM, Paxton RJ, Soro A (2021). Urbanization is associated with shifts in bumblebee body size, with cascading effects on pollination. Evol Appl.

[CR52] Therneau T (2020a) A Package for Survival Analysis in R_. R package version 3.2-7. https://CRAN.R-project.org/package=survival

[CR53] Therneau TM (2020b) coxme: Mixed Effects Cox Models. R package version 2.2-16. https://CRAN.R-project.org/package=coxme

[CR54] Troen AM, French EE, Roberts JF, Selhub J, Ordovas JM, Parnell LD, Lai CQ (2007). Lifespan modification by glucose and methionine in *Drosophila melanogaster* fed a chemically defined diet. Age.

[CR55] Vaudo AD, Tooker JF, Grozinger CM, Patch HM (2015). Bee nutrition and floral resource restoration. Curr Opin Insect Sci.

[CR56] Vaudo AD, Patch HM, Mortensen DA, Tooker JF, Grozinger CM (2016). Macronutrient ratios in pollen shape bumble bee (*Bombus impatiens*) foraging strategies and floral preferences. Proc Natl Acad Sci.

[CR57] Vaudo AD, Stabler D, Patch HM, Tooker JF, Grozinger CM, Wright GA (2016). Bumble bees regulate their intake of essential protein and lipid pollen macronutrients. J Exp Biol.

[CR58] Vaudo AD, Farrell LM, Patch HM, Grozinger CM, Tooker JF (2018). Consistent pollen nutritional intake drives bumble bee (*Bombus impatiens*) colony growth and reproduction across different habitats. Ecol Evol.

[CR59] Vaudo AD, Tooker JF, Patch HM, Biddinger DJ, Coccia M, Crone MK, Fiely M, Francis JS, Hines HM, Hodges M (2020). Pollen protein: lipid macronutrient ratios may guide broad patterns of bee species floral preferences. Insects.

[CR60] Wickham H (2016). ggplot2: elegant graphics for data analysis.

[CR61] Wilfert L, Brown MJ, Doublet V (2020). OneHealth implications of infectious diseases of wild and managed bees. J Invert Pathol.

[CR62] Wolschin F, Shpigler H, Amdam GV, Bloch G (2012). Size-related variation in protein abundance in the brain and abdominal tissue of bumble bee workers. Insect Mol Biol.

[CR63] Worden BD, Skemp AK, Papaj DR (2005). Learning in two contexts: the effects of interference and body size in bumblebees. J Exp Biol.

[CR64] Yerushalmi S, Bodenhaimer S, Bloch G (2006). Developmentally determined attenuation in circadian rhythms links chronobiology to social organization in bees. J Exp Biol.

