# Supplementary material for: Complex relationship between amino acids, fitness and food intake in Bombus terrestris
Source: Amino Acids. 2021 Sep 29;53(10):1545–58. doi: 10.1007/s00726-021-03075-8 (PMC8519840; doi:10.1007/s00726-021-03075-8)
Supplement: Supplementary file 1 — Supplementary file1 (DOCX 2348 kb) [file 726_2021_3075_MOESM1_ESM.docx]

**SUPPLEMENTARY INFORMATION - COMPLEX RELATIONSHIP BETWEEN AMINO ACIDS, FITNESS AND FOOD INTAKE IN BOMBUS TERRESTRIS**

C. Ruth Archer^a^*****, Johannes Fähnle^a^, Maximilian Pretzner^a^, Cansu Üstüner^a^, Nina Weber^a^, Andreas Sutter^b^, Vincent Doublet^a^, Lena Wilfert^a,c^

1. Institute of Evolutionary Ecology and Conservation Genomics, University of Ulm

Albert-Einstein-Allee 11, 89081 Ulm, Germany

1. School of Biological Sciences, University of East Anglia, Norwich, UK.
2. College of Life and Environment Sciences, Tremough Campus, University of Exeter, Penryn, UK. TR10 8FL.

**CONTENTS**

Table S1. Proportions of AAs used in our “pollen” AA mix.

Table S2. Evaporative water loss associated with each AA:C ratio, and AA mix.

Table S3. Summary of Experimental Design

Table S4. Sample Sizes.

Table S5. DIC values for each Bayesian model used in the model selection process.

Figure S1. Location of the marginal cell used to control for any variation in bee size.

Figure S2. Example image of each ovarian activation level.

Figure S3. Relationship between wing length and lipid mass.

Figure S4. Relationship between wing length and abdomen dry mass.

**Table S1. Proportions of each AA in the pollen collected by bumble bees in Leonard and Blüthgen (2012).** Because the 10 AAs used in the current study amounted to 38% of the AAs in the bumble bee collected pollen in Leonard and Blüthgen (2012), we created diets where the relative proportion of these AAs to one another was identical to that in Leonard and Blüthgen (2012) but they sum to 1.

| **Amino Acid** | **Proportion in full pollen** | **Proportion in pollen AA mix** |
| --- | --- | --- |
| Methionine | 0.017 | 0.0445 |
| Tryptophan | 0.002 | 0.0052 |
| Leucine | 0.077 | 0.2016 |
| Lysine | 0.0575 | 0.1505 |
| Valine | 0.0455 | 0.1191 |
| Arginine | 0.0395 | 0.1034 |
| Isoleucine | 0.0325 | 0.08514 |
| Phenylalanine | 0.0375 | 0.0982 |
| Threonine | 0.053 | 0.1387 |
| Histidine | 0.0205 | 0.0537 |
| Total | 0.382 | 1 |

**Table S2**. Evaporative water loss associated with each AA:C ratio, and AA mix.

| **Diet AA:C Ratio** | **Evaporative Loss – g** | |
| --- | --- | --- |
|  | **Pollen AA Mix** | **Equimolar AA Mix** |
| 0:1 | 0.222 ± 0.02 | 0.236 ± 0.05 |
| 1:250 | 0.197 ± 0.01 | 0.238 ± 0.06 |
| 1:100 | 0.198 ± 0.03 | 0.192 ± 0.05 |
| 1:75 | 0.220 ± 0.02 | 0.218 ± 0.05 |
| 1:50 | 0.222 ± 0.04 | 0.213 ± 0.06 |
| 1:25 | 0.168 ± 0.01 | 0.247 ± 0.06 |
| 1:10 | 0.204 ± 0.02 | 0.226 ± 0.07 |

**Table S3. Summary of Experimental Design.** This table is intended to provide an overview of the dietary manipulation treatments and which traits (shaded in green) were measured, on each experimental diet.

|  | **AA Mix** | **Pollen** | | | | | | | **Equimolar** | | | | | | |
| --- | --- | --- | --- | --- | --- | --- | --- | --- | --- | --- | --- | --- | --- | --- | --- |
|  | **AA:C Ratio** | 0:1 | 1:250 | 1:100 | 1:75 | 1:50 | 1:25 | 1:10 | 0:1 | 1:250 | 1:100 | 1:75 | 1:50 | 1:25 | 1:10 |
| **Trait** | Intake Array |  |  |  |  |  |  |  |  |  |  |  |  |  |  |
|  | Survival during feeding |  |  |  |  |  |  |  |  |  |  |  |  |  |  |
|  | Abdomen Composition |  |  |  |  |  |  |  |  |  |  |  |  |  |  |
|  | Survival following food removal |  |  |  |  |  |  |  |  |  |  |  |  |  |  |
|  | Ovarian Activation |  |  |  |  |  |  |  |  |  |  |  |  |  |  |

**Table S4.** The number of bees assayed for each trait.

| **Trait** | **AA:C Ratio** | | | | | | |
| --- | --- | --- | --- | --- | --- | --- | --- |
|  | **0:1** | **1:250** | **1:100** | **1:75** | **1:50** | **1:25** | **1:10** |
| Abdomen Composition | 21 | 17 | 20 | 22 | 22 | 22 | 22 |
| Survival following food removal | 24 | 22 | 20 | 24 | 23 | 22 | 24 |
| Ovarian Activation – Pollen mix | 25 | 25 | 25 | 25 | 25 | 25 | 25 |
| Ovarian Activation – Equimolar mix | 25 | 24 | 25 | 23 | 22 | 24 | 22 |

**Table S5. DIC Bayesian models.** Comparison of multinomial models created using MCMCglmm ranging from the most (1) to least (5) complex.

| **Model** | **Explanatory variables** | **DIC** |
| --- | --- | --- |
| 1 | AA:C + AA Mix + AA:C:AA Mix | 697.21 |
| 2 | AA:C + AA Mix | 700.69 |
| 3 | AA:C | 713.23 |
| 4 | AA Mix | 694.20 |
| 5 | Null | 692.72 |

**Figure S1. Location of the marginal cell used to measure individual bee size.**

*
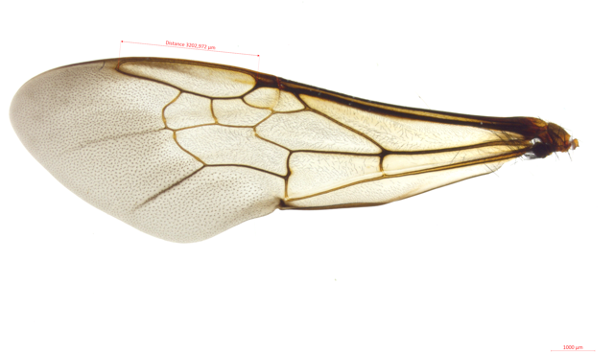
*

**Figure S2. Example ovarian activation levels.** Ovaries in panel A are inactive, ovaries in panel B are classed as showing intermediate activation, while those in panel C are classed as fully activated.

*
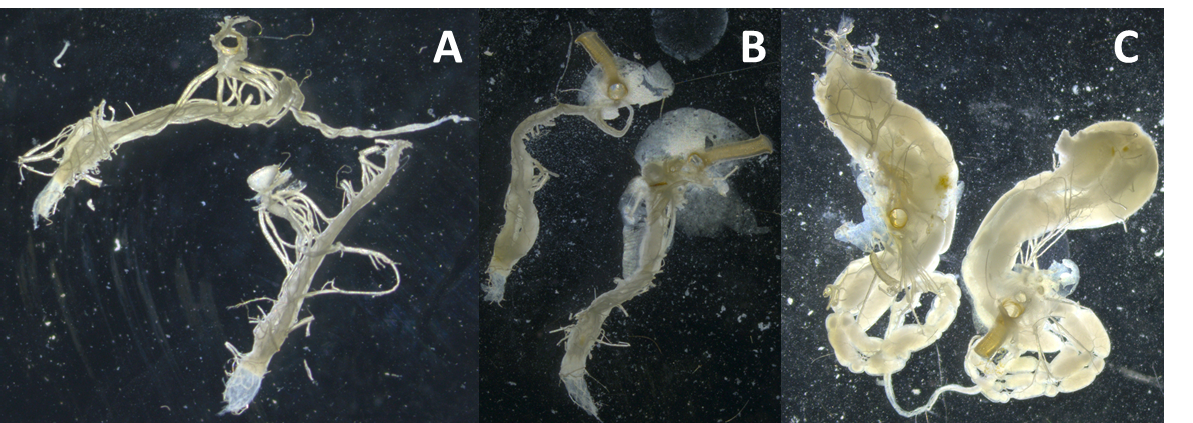
*

**Figure S3. Relationship between wing length and lipid mass.** Lines show the relationship between wing length and lipid mass for each individual micro-colony.


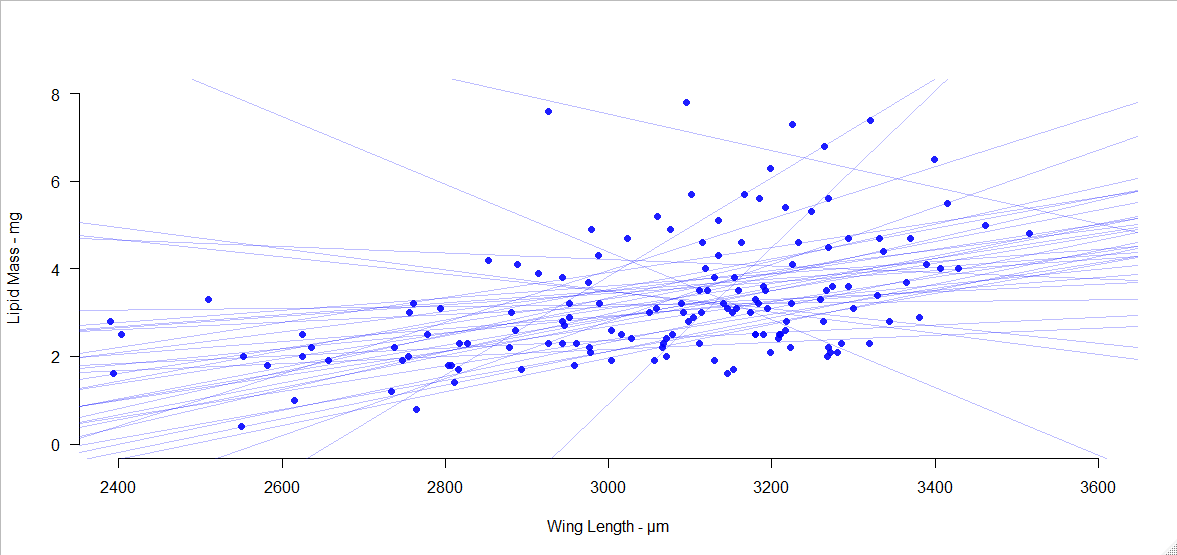


**Figure S4. Relationship between wing length and abdomen dry mass – excluding lipids.** Lines show the relationship between wing length and abdomen dry mass for each individual micro-colony.


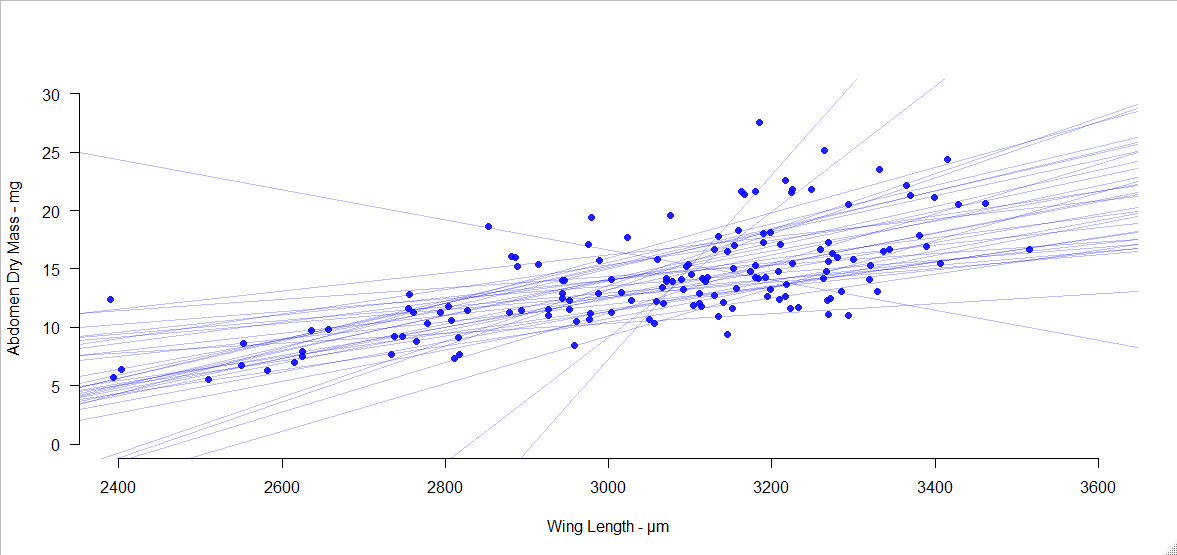


**References**

Leonhardt SD, Blüthgen N (2012) The same, but different: pollen foraging in honeybee and bumblebee colonies. Apidologie 43:449–464. https://doi.org/10.1007/s13592-011-0112-y
